# Supplementary material for: Periostin interaction with discoidin domain receptor-1 (DDR1) promotes cartilage degeneration
Source: PLoS One. 2020 Apr 24;15(4):e0231501. doi: 10.1371/journal.pone.0231501 (PMC7182230; doi:10.1371/journal.pone.0231501)
Supplement: S1 Fig — (PPTX) [file pone.0231501.s001.pptx]

## Slide 1
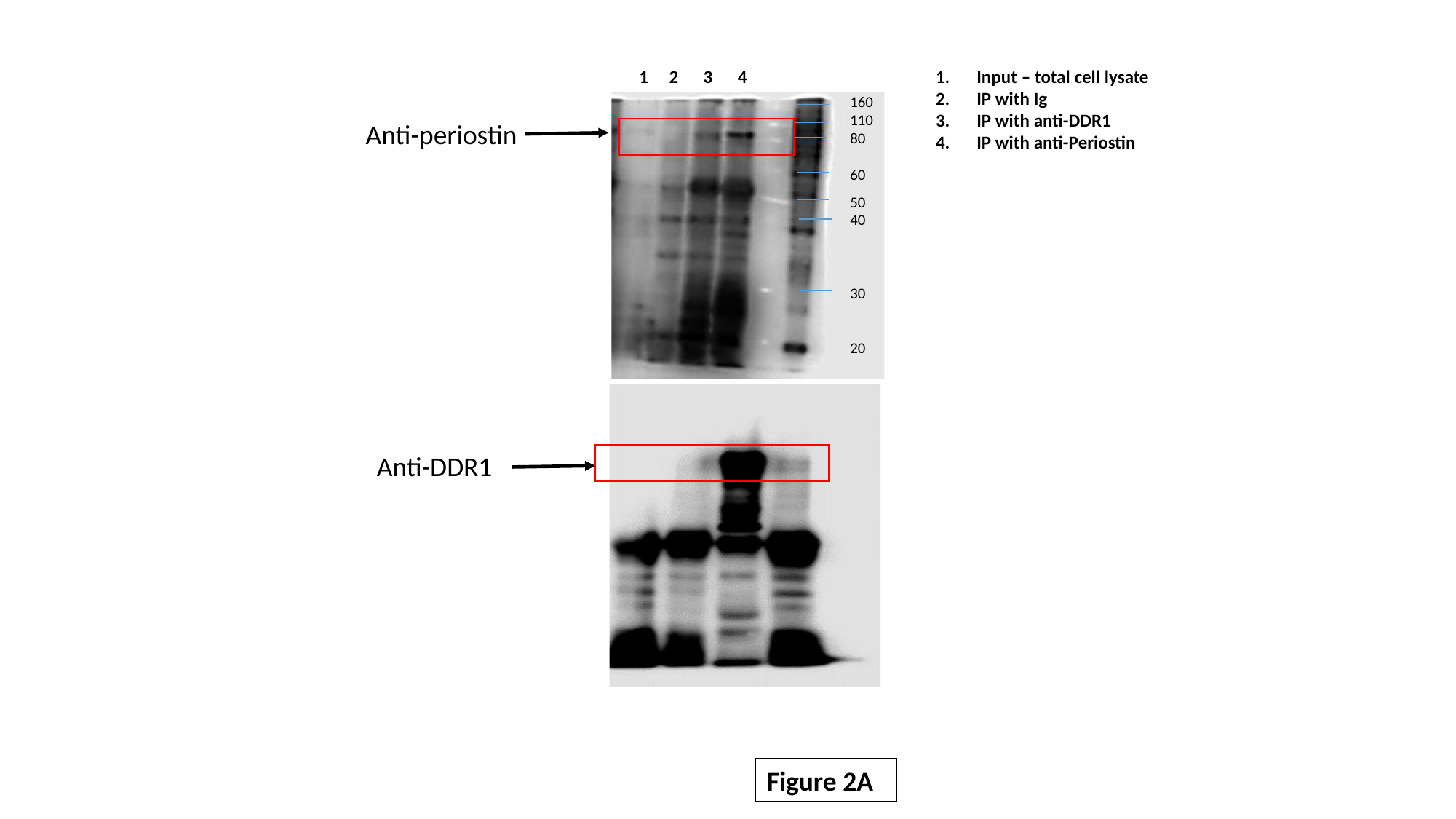

1 2 3 4
Input – total cell lysate
IP with Ig
IP with anti-DDR1
IP with anti-Periostin
160
110
80
60
50
40
30
20
Anti-periostin
Anti-DDR1
Figure 2A

## Slide 2
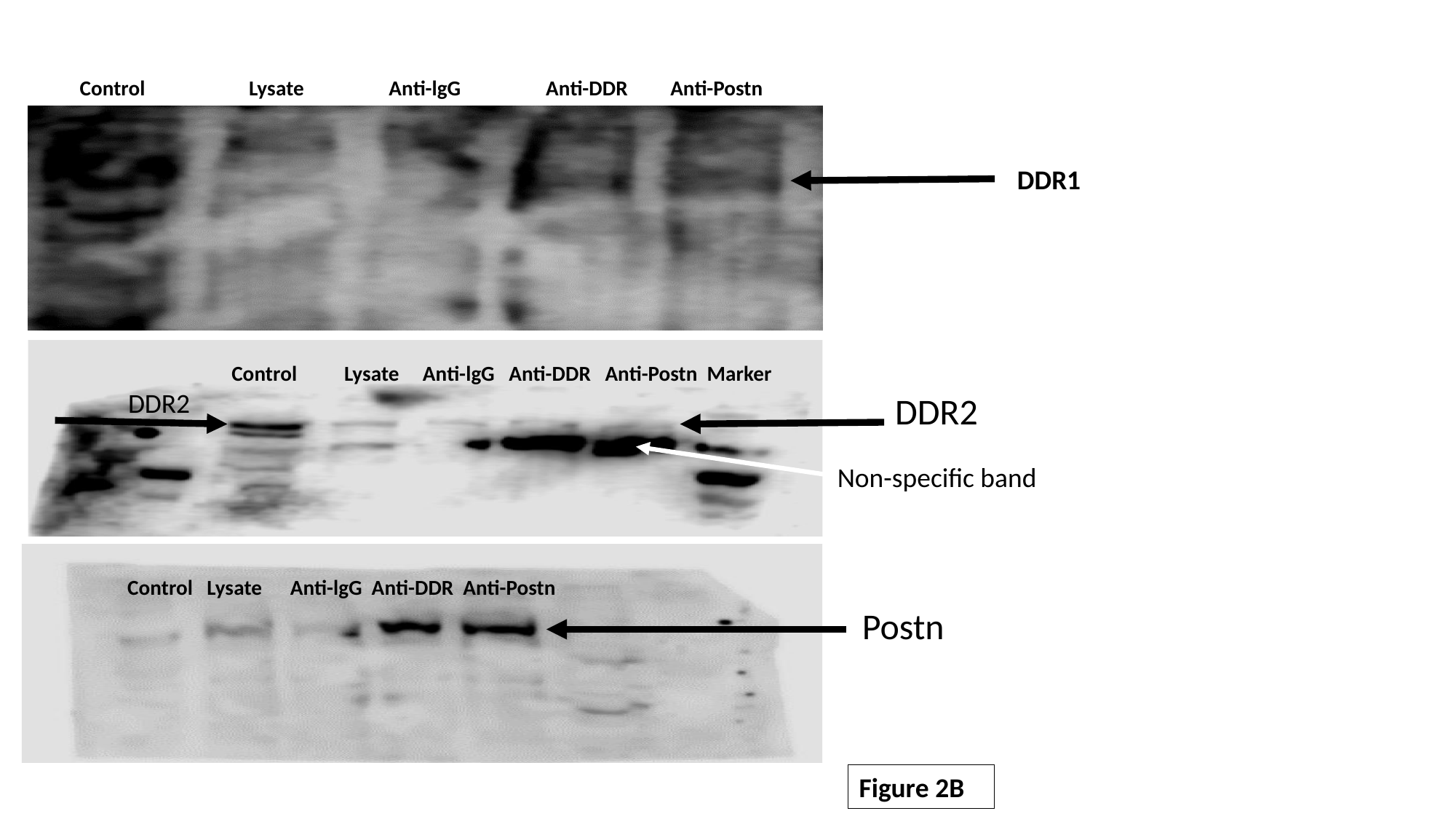

Control Lysate Anti-lgG Anti-DDR Anti-Postn
DDR1
 Control Lysate Anti-lgG Anti-DDR Anti-Postn Marker
DDR2
DDR2
Non-specific band
Control Lysate Anti-lgG Anti-DDR Anti-Postn
Postn
Figure 2B

## Slide 3
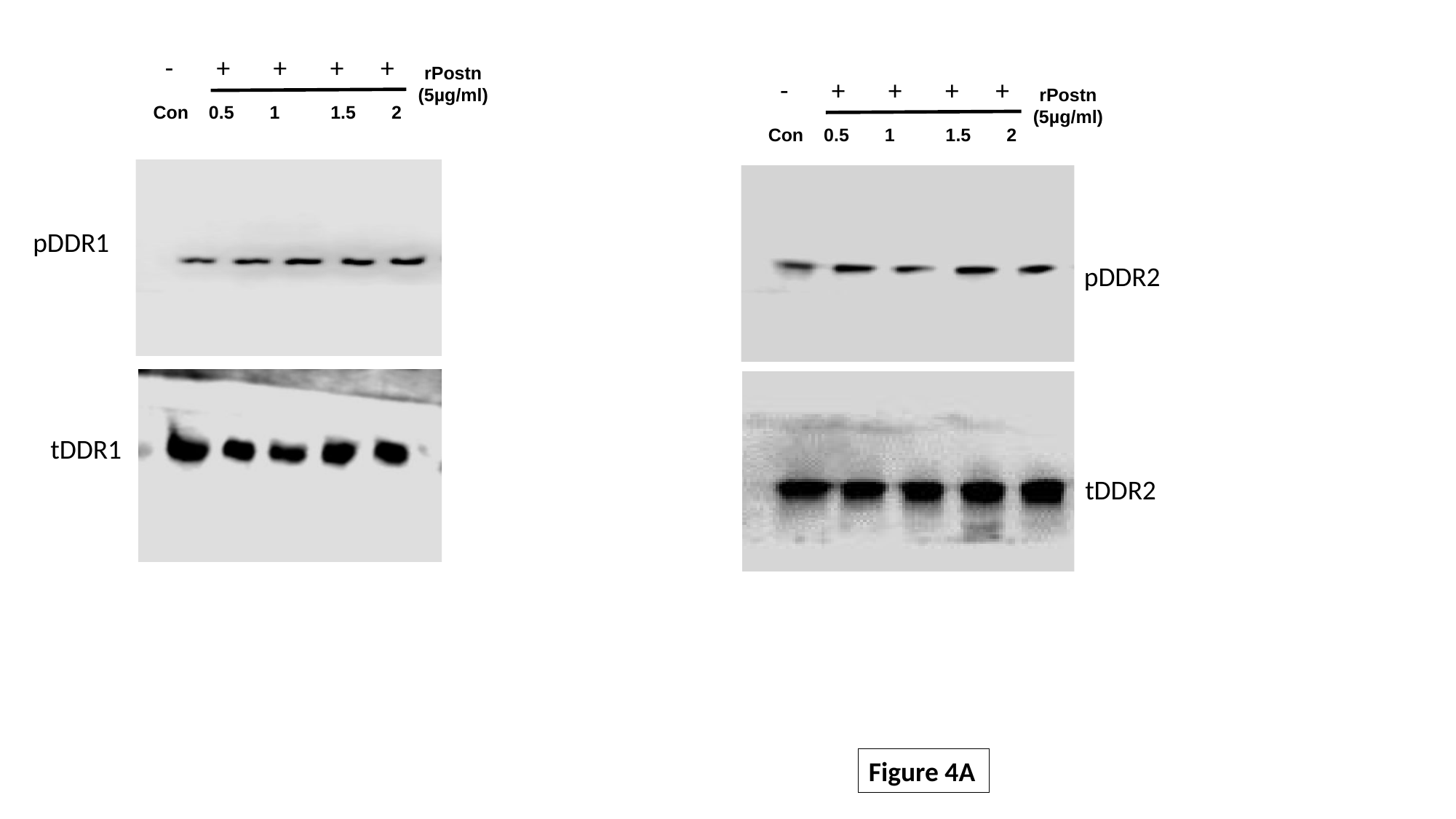

- + + + +
rPostn (5µg/ml)
 Con 0.5 1 1.5 2
pDDR1
tDDR1
- + + + +
rPostn (5µg/ml)
 Con 0.5 1 1.5 2
pDDR2
tDDR2
Figure 4A

## Slide 4
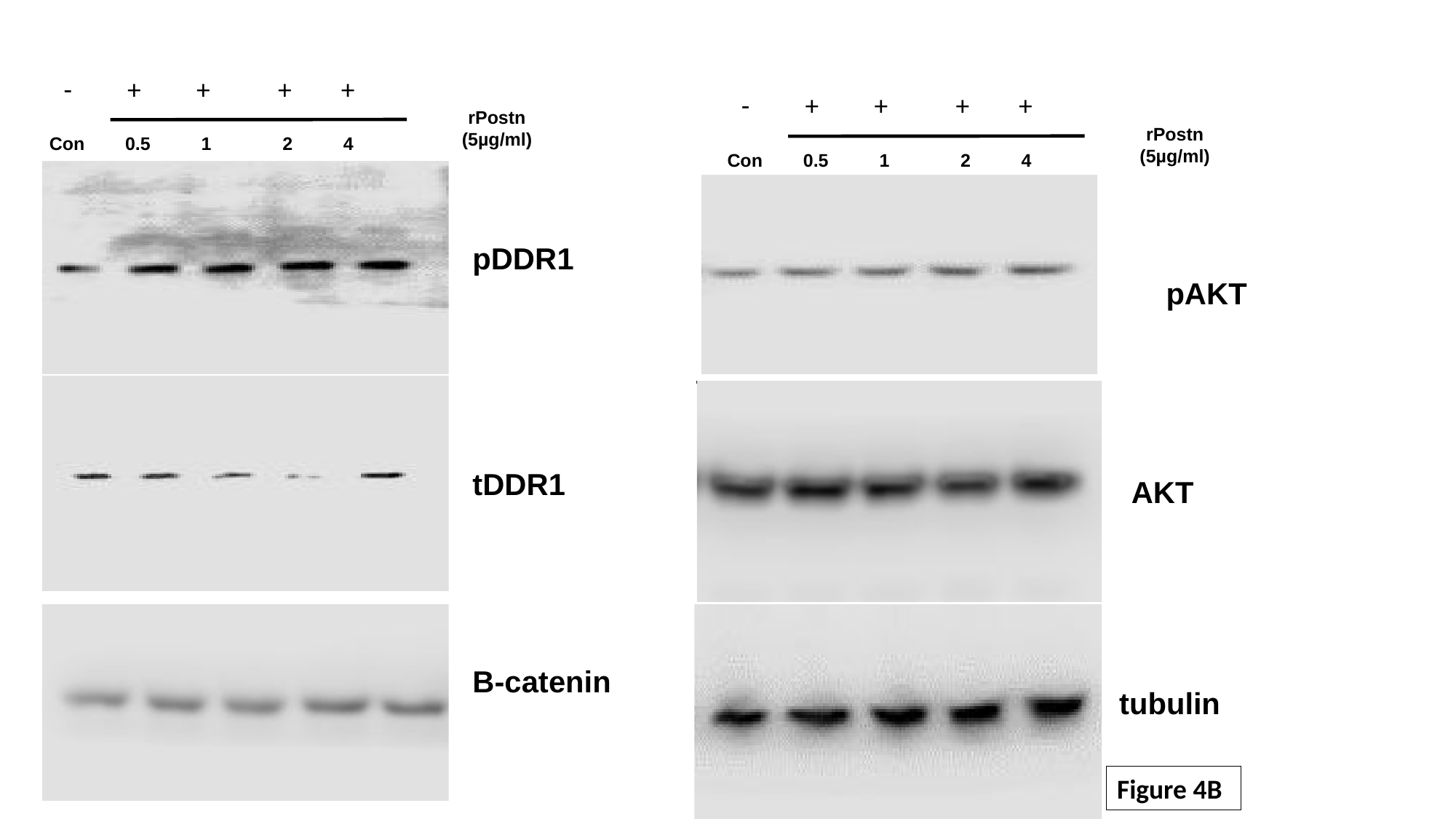

- + + + +
- + + + +
rPostn (5µg/ml)
rPostn (5µg/ml)
 Con 0.5 1 2 4
 Con 0.5 1 2 4
pDDR1
pAKT
tDDR1
AKT
B-catenin
tubulin
Figure 4B

## Slide 5
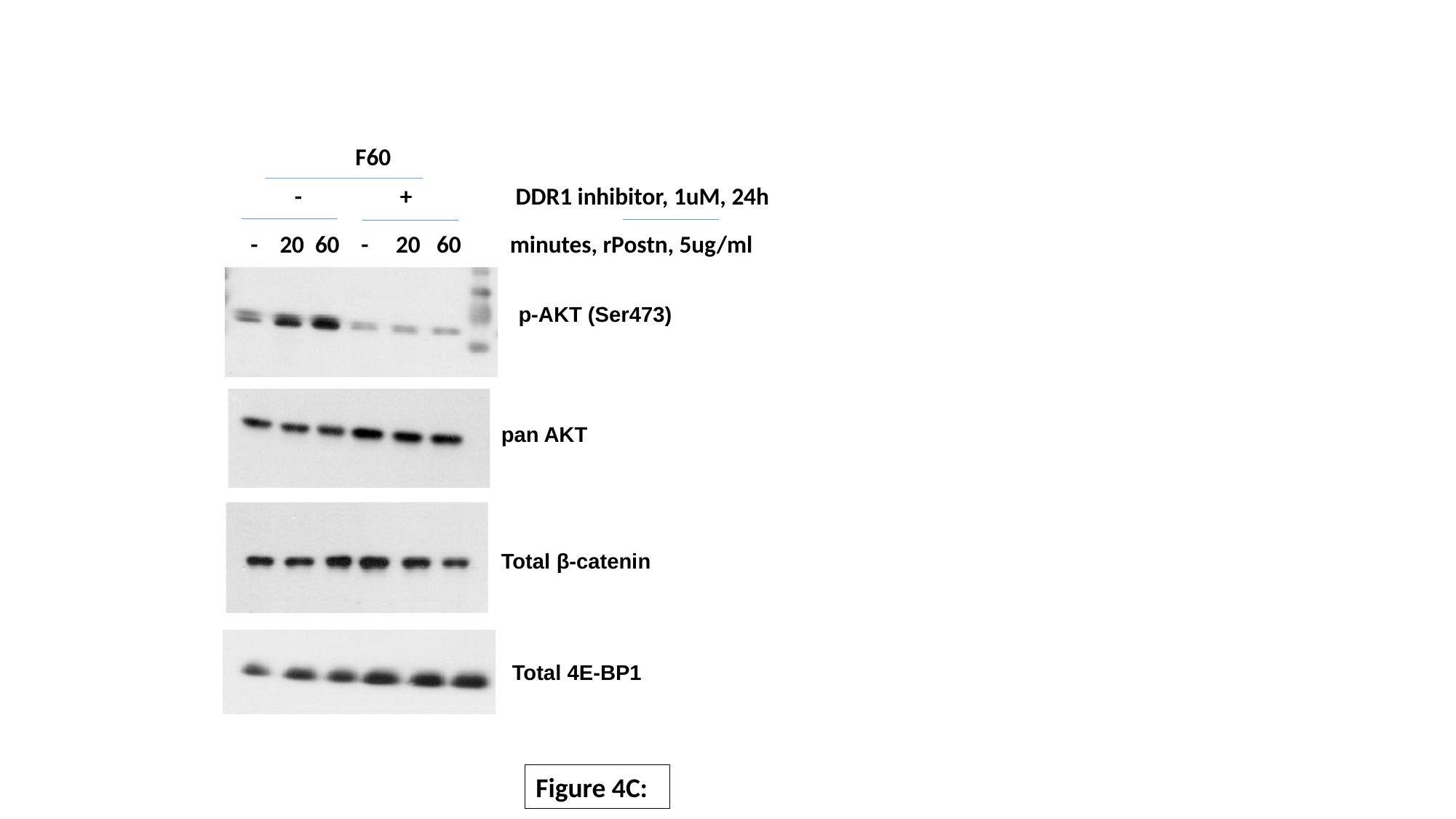

F60
 - + DDR1 inhibitor, 1uM, 24h
p-AKT (Ser473)
pan AKT
Total β-catenin
Total 4E-BP1
 - 20 60 - 20 60 minutes, rPostn, 5ug/ml
Figure 4C:
